# W. Lawson Tait on the Modern Treatment of Uterine Myoma

**Published:** 1885-09

**Authors:** 


### MR. LAWSON TAIT ON THE MODERN TREATMENT OF UTERINE MYOMA.

MR. LAWSON TAIT'S recent paper, entitled "*The Modern Treatment of Uterine Myoma*," read in the section of Obstetric Medicine at the annual meeting of the British Medical Association in Cardiff, will attract universal attention. This paper supplements the discussion of DR. THOMAS MORE MADDEN'S essay upon the subject before the British Gynecological Society on Wednesday, 27th May, 1885.

In this discussion MR. TAIT said, with reference to the management of uterine myoma, "I must not go into the question of medical treatment, because it is very much like the chapter in the History of Ireland about snakes—there are no snakes in Ireland. The medical treatment of uterine myomata is a myth."

MR. TAIT'S modern experience confirms the conclusions of his paper, read 24th May, 1881, before the Royal Medical and Chirurgical Society, and published in *The American Journal of the Medical Sciences*, January, 1882.

The first point of his thesis is "to show that removal of the uterine appendages for myoma, when properly performed, is not a fatal operation, but one with hardly any mortality at all, even when the tumours are large, and when the patients are brought almost to death's door by hæmorrhage."

In support of this proposition, a list of fifty-eight operations without a single death, since January, 1884, is appended.

The second point in the thesis is: "That the results of oöphorectomy are satisfactory and permanent, so that we may with confidence recommend it for relief of suffering and the saving of life."

In support of this proposition, statements as to the present condition of his first fifty cases, drawn from personal observation, the practitioner who sent the case, or the patient herself, are adduced. Of the fifty cases, failure occurred only in two instances.

"Somewhat jealous for the honor of English surgery,' MR. TAIT claims the priority of the introduction of oöphorectomy. He proposed and discussed the procedure in October, 1871, and successfully performed the operation on February 11th, 1872. HEGAR'S first operation, which was unsuccessful, was performed July 27th, 1872.

MR. TAIT'S second successful operation was performed on August 1st, 1872.

DR. BATTEY'S first operation, which was also successful, was performed on August 17th, 1872.

